# Acidic mammalian chitinase is not a critical target for allergic airway disease in mice

**DOI:** 10.1186/1476-9255-10-S1-P5

**Published:** 2013-08-14

**Authors:** LJ Fitz, C DeClercq, J Brooks, W Kuang, B Bates, D Demers, A Winkler, K Nocka, A Jiao, RM Greco, LE Mason, M Fleming, A Quazi, J Wright, S Goldman, C Hubeau, CM Williams

**Affiliations:** 1 Inflammation and Immunology, Drug Safety R&D, Pfizer Cambridge, Massachusetts, 02140, USA

## 

The expression of acidic mammalian chitinase (AMCase) is associated with Th2-driven respiratory disorders. To investigate the potentially pathological role of AMCase in allergic airway disease (AAD), we sensitized and challenged mice with ovalbumin or a combination of house dust mite (HDM) plus cockroach allergen. These mice were treated or not treated with small molecule inhibitors of AMCase, which significantly reduced allergen-induced chitinolytic activity in the airways, but exerted no apparent effect on pulmonary inflammation per se. Transgenic and AMCase-deficient mice were also submitted to protocols of allergen sensitization and challenge, yet we found little or no difference in the pattern of AAD between mutant mice and wild-type (WT) control mice. In a separate model, where mice were challenged only with intratracheal instillations of HDM without adjuvant, total bronchoalveolar lavage (BAL) cellularity, inflammatory infiltrates in lung tissues, and lung mechanics remained comparable between AMCase-deficient mice and WT control mice. However BAL neutrophil and lymphocyte counts were significantly increased in AMCase-deficient mice, whereas concentrations in BAL of IL-13 were significantly decreased compared with WT control mice. These results indicate that, although exposure to allergen stimulates the expression of AMCase and increased chitinolytic activity in murine airways, the overexpression or inhibition of AMCase exerts only a subtle impact on AAD. Conversely, the increased numbers of neutrophils and lymphocytes in BAL and the decreased concentrations of IL-13 in AMCase-deficient mice challenged intratracheally with HDM indicate that AMCase contributes to the Th1/Th2 balance in the lungs. This finding may be of particular relevance to patients with asthma and increased airway neutrophilia.

